# Severe *Streptococcus equi* Subspecies *zooepidemicus* Outbreak from Unpasteurized Dairy Product Consumption, Italy

**DOI:** 10.3201/eid2905.221338

**Published:** 2023-05

**Authors:** Serena Bosica, Alexandra Chiaverini, Maria Elisabetta De Angelis, Antonio Petrini, Daniela Averaimo, Michele Martino, Marco Rulli, Maria Antonietta Saletti, Maria Chiara Cantelmi, Franco Ruggeri, Fabrizio Lodi, Paolo Calistri, Francesca Cito, Cesare Cammà, Marco Di Domenico, Antonio Rinaldi, Paolo Fazii, Fabrizio Cedrone, Giuseppe Di Martino, Patrizia Accorsi, Daniela Morelli, Nicola De Luca, Francesco Pomilio, Giustino Parruti, Giovanni Savini

**Affiliations:** Istituto Zooprofilattico Sperimentale Abruzzo e Molise “G. Caporale,” Teramo, Italy (S. Bosica, A. Chiaverini, M.E. De Angelis, A. Petrini, D. Aberaimo, M. Martino, M. Rulli, M.A. Saletti, M.C. Cantemi, P. Calistri, F. Cito, C. Cammà, M. Di Domenico, A. Rinaldi, D. Morelli, F. Pomilio, G. Savini);; University of Teramo, Teramo (M.C. Cantelmi, F. Cito);; Azienda USL di Pescara, Pescara, Italy (F. Ruggeri, F. Lodi, G. Di Martino, P. Accorsi, N. De Luca);; Presidio Ospedaliero “Santo Spirito,” Pescara (P. Fazii, F. Cedrone, G. Parruti)

**Keywords:** *Streptococcus equi* subspecies *zooepidemicus*, bacteria, food safety, meningitis/encephalitis, streptococci, zoonosis, septicemia, arthritis, foodborne outbreak, raw milk dairy products, whole genome sequencing, Italy

## Abstract

During November 2021–May 2022, we identified 37 clinical cases of *Streptococcus equi* subspecies *zooepidemicus* infections in central Italy. Epidemiologic investigations and whole-genome sequencing showed unpasteurized fresh dairy products were the outbreak source. Early diagnosis by using sequencing technology prevented the spread of life-threatening *S. equi* subsp. *zooepidemicus* infections.

*Streptococcus* *equi* subspecies *zooepidemicus* is a β-hemolytic streptococcus expressing Lancefield group C antigen and is 1 of 3 *S. equi* subspecies. *S.* *equi* subsp. *zooepidemicus* is an opportunistic pathogen that can infect domestic animals, pets, and wildlife (*1*–*6*). Sporadic human cases have been reported (*7*), characterized by clinical manifestations that vary from meningitis to sepsis. Human infection generally occurs through direct contact with infected animals or by consumption of contaminated unpasteurized milk or other dairy products (*8*–*10*). We report a large *S.* *equi* subsp. *zooepidemicus* outbreak in Italy.

## The Study

**Figure 1:**
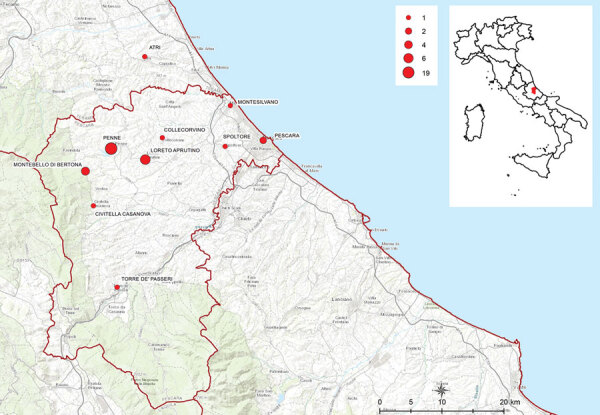
Location of residential areas in the province of Pescara and neighboring zones where outbreak cases were found in study of severe *Streptococcus equi* subspecies *zooepidemicus* outbreak from unpasteurized dairy product consumption, Italy, during November 2021–May 2022. Sizes of red dots indicate numbers (N = 37) of patients infected by location. Inset shows the outbreak area in the Vestina region of central Italy.

During November 2021–February 2022, *S.* *equi* subsp. *zooepidemicus* infections were detected in 18 hospitalized patients who resided in a limited area within the province of Pescara, Italy (Figure 1). A wide range of clinical manifestations were observed in the patients, including septicemia, pharyngitis, arthritis, uveitis, and endocarditis. Five of those patients died from severe meningitis. For most cases, *S.* *equi* subsp. *zooepidemicus* bacteremia was accompanied by >1 additional localized bacterial site. *S.* *equi* subsp. *zooepidemicus* was detected in various patient specimens, including articular effusions and cerebrospinal fluid, that were tested by hospital laboratories. A task force consisting of physicians, veterinarians, epidemiologists, scientists, microbiologists, and communication experts was created and coordinated by the local competent authority to evaluate the outbreak. 

We conducted an active investigation of *S.* *equi* subsp. *zooepidemicus* cases, defined as patients with laboratory-confirmed infection. We identified 37 outbreak cases over a 7-month period. We completed interviews of 36 patients or their relatives by using telephone-administered questionnaires. Patients were 6–98 (median 79) years of age. Male (n = 16) and female (n = 21) patients were similarly represented (Table 1). We obtained 21 bacteria isolates from 19 hospitalized patients that were sent to Istituto Zooprofilattico Sperimentale in Teramo, Italy. We identified bacteria species by using a Biotyper matrix-assisted laser desorption/ionization time-of-flight mass spectrometer (Bruker, https://www.bruker.com).

**Table 1:** Patient characteristics in study of severe *Streptococcus equi* subspecies *zooepidemicus* outbreak from unpasteurized dairy product consumption in central Italy during November 2021–May 2022*

Characteristics	Value
Total no. patients	37
Patients with symptoms	35 (94.6)
Hospitalized patients	23 (62.2)
Patient deaths†	5 (13.5)
Male patients	16 (43.2)
Age range (median), y	6–98 (79)
Age group, y	
<15	1
16–39	1
40–59	4
60–79	12
>80	19
Symptoms	
Fever	27 (75)
Diarrhea	6 (16.7)
Headache	5 (13.9)
Vomiting	4 (11.1)
Septicemia	3 (8.3)
Cystitis	2 (5.6)
Pharyngitis	2 (5.6)

*Values are no. (%) patients except as indicated. †Number of hospitalized patients who died.

We performed whole-genome sequencing for 21 isolates by using the Illumina platform (Illumina, https://www.illumina.com) with a setting of 300 cycles (150-bp paired-end reads) (*11*). After read quality checks, we confirmed all clinical strains were *S.* *equi* subsp. *zooepidemicus* by using the KmerFinder tool (*12*). We assigned sequence type (ST) 61 to trains by using a multilocus sequence typing scheme (*13*) (https://pubmlst.org/streptococcus-zooepidemicus). 

We confirmed correlations between human strains by performing single-nucleotide polymorphism (SNP) analysis through the US Food and Drug Administration Center for Food Safety and Applied Nutrition pipeline (*14*) and by using MinION technology (*15*) to obtain a hybrid genome from an outbreak strain as a reference. We found that all 21 clinical strains were closely related (0–3 SNPs), implying involvement of a unique source strain in the observed human cases. Moreover, the antimicrobial susceptibility test performed on all human strains gave identical results, confirming the hypothesis that 1 source strain was responsible for all human cases.

An extensive investigation was performed by public health, veterinary, and food hygiene services to identify the infection source. Epidemiologic analysis showed that 31 patients consumed soft or semi-soft cheeses purchased from local producers or dealers. A total of 8 local dairy food business operators were inspected. Samples of raw bulk milk (from cows and sheep), fresh cheese from unpasteurized and pasteurized milk, and water were obtained from each operator and sent to Istituto Zooprofilattico Sperimentale, Teramo, for bacteria detection. We cultured the samples and isolated *Streptococcus* spp. from sheep blood agar after incubation for 24 ±1 h at 37°C ±1°C in a 5% CO_2_ enriched atmosphere. We tested all samples by using Genesig real-time PCR kits (Primerdesign Ltd, http://www. primerdesign.co.uk). We performed species identification and whole-genome sequencing as described for human samples. We detected *S.* *equi* subsp. *zooepidemicus* in an unpasteurized bulk cow milk sample taken from 1 dairy producer selected within the outbreak area. That operator was then officially inspected and sampled by local competent authorities; a total of 18 *S.* *equi* subsp. *zooepidemicus* strains were isolated from 2 bulk milk tanks and 2 cured raw milk cheese samples. Genome sequences from those samples were also ST61; SNP analysis showed the 18 strains clustered with the clinical strains, indicating a strong correlation between the operator- and human-derived strains.

**Figure 2:**
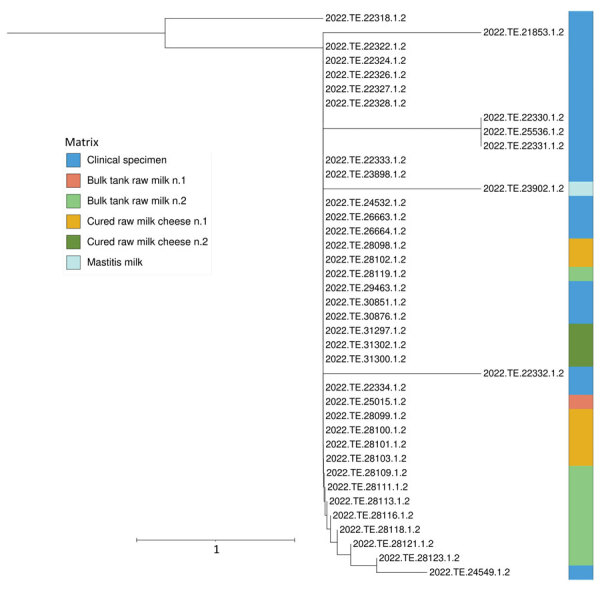
Phylogenetic analysis of *Streptococcus equi* subspecies *zooepidemicus* strains in study of severe outbreak from unpasteurized dairy product consumption, Italy. We performed whole-genome sequencing and single-nucleotide polymorphism analysis of 40 *S. equi* subsp. *zooepidemicus* strains isolated from clinical and dairy food samples (Table 2). Tree was generated by using the neighbor-joining method. Different colors indicate the different types of samples. Strains were isolated from 2 samples each taken from bulk tank raw milk and cured raw milk cheese. Tree shows clinical specimen sequences clustered with those from dairy products. Scale bar indicates nucleotide substitutions per site.

After *S.* *equi* subsp. *zooepidemicus* was identified as the pathogen responsible for the human cases, we reviewed data from the Istituto Zooprofilattico Sperimentale strain library. We found a strain that was isolated from the milk of a cow with mastitis in November 2021; the animal belonged to the same operator whose products tested positive. After sequencing, the strain was also identified as ST61 (Table 2) and clustered with the other trains isolated from human patients and raw milk products (Figure 2).

**Table 2:** Metadata of 40 *Streptococcus equi* subspecies *zooepidemicus* strains typed by whole genome sequencing in study of severe outbreak from unpasteurized dairy product consumption, Italy*

Identification no.	Matrix†
2022.TE.21853.1.2	Clinical specimen
2022.TE.22318.1.2	Clinical specimen
2022.TE.22322.1.2	Clinical specimen
2022.TE.22324.1.2	Clinical specimen
2022.TE.22326.1.2	Clinical specimen
2022.TE.22327.1.2	Clinical specimen
2022.TE.22328.1.2	Clinical specimen
2022.TE.22330.1.2	Clinical specimen
2022.TE.22331.1.2	Clinical specimen
2022.TE.22332.1.2	Clinical specimen
2022.TE.22333.1.2	Clinical specimen
2022.TE.22334.1.2	Clinical specimen
2022.TE.23898.1.2	Clinical specimen
2022.TE.23902.1.2	Mastitis milk
2022.TE.24532.1.2	Clinical specimen
2022.TE.24549.1.2	Clinical specimen
2022.TE.25015.1.2	Bulk tank raw milk no. 1
2022.TE.25536.1.2	Clinical specimen
2022.TE.26663.1.2	Clinical specimen
2022.TE.26664.1.2	Clinical specimen
2022.TE.28098.1.2	Cured raw milk cheese no. 1
2022.TE.28099.1.2	Cured raw milk cheese no. 1
2022.TE.28100.1.2	Cured raw milk cheese no. 1
2022.TE.28101.1.2	Cured raw milk cheese no. 1
2022.TE.28102.1.2	Cured raw milk cheese no. 1
2022.TE.28103.1.2	Cured raw milk cheese no. 1
2022.TE.28109.1.2	Bulk tank raw milk no. 2
2022.TE.28111.1.2	Bulk tank raw milk no. 2
2022.TE.28113.1.2	Bulk tank raw milk no. 2
2022.TE.28116.1.2	Bulk tank raw milk no. 2
2022.TE.28118.1.2	Bulk tank raw milk no. 2
2022.TE.28119.1.2	Bulk tank raw milk no. 2
2022.TE.28121.1.2	Bulk tank raw milk no. 2
2022.TE.28123.1.2	Bulk tank raw milk no. 2
2022.TE.29463.1.2	Clinical specimen
2022.TE.30851.1.2	Clinical specimen
2022.TE.30876.1.2	Clinical specimen
2022.TE.31297.1.2	Cured raw milk cheese no. 2
2022.TE.31300.1.2	Cured raw milk cheese no. 2
2022.TE.31302.1.2	Cured raw milk cheese no. 2

*All 40 samples were sequence type 61. †Strains were isolated from 2 samples each (nos. 1 and 2) taken from bulk tank raw milk and cured raw milk cheese.

On the basis of epidemiologic and laboratory analyses, local competent authorities established measures to limit and prevent *S.* *equi* subsp. *zooepidemicus* spread by the end of February 2022. All dairy products were recalled from the market and local dealers and ripening cheeses were destroyed; local authorities required pasteurization of milk intended for cheese production.

## Conclusions

We report epidemiologic, microbiologic, and genomic findings from a *S.* *equi* subsp. *zooepidemicus* outbreak that involved 37 patients in Italy. We found strong genomic correlation between strains isolated from case-patients, unpasteurized milk and dairy products, and milk from infected cows that clearly indicated a zoonotic infection source. Questionnaire and laboratory data showed that human infections were caused by consuming unpasteurized fresh cheese produced from infected milk cows. We were unable to trace the origin of infection back to specific dairy food business operator livestock. The farmer who had the positive cow referred to construction work in the barn during October–November 2021, which might have caused stress, predisposing the animal to mastitis. A possible reactivation of a latent *S.* *equi* subsp. *zooepidemicus* infection cannot be excluded. Identifying the source of infection in a relatively short time enabled a rapid response that prevented further cases in the community. However, the outbreak in Italy was considered the largest and most severe outbreak associated with the consumption of unpasteurized fresh cheese that has been reported.

In summary, our study implicates *S.* *equi* subsp. *zooepidemicus* as a possible zoonotic pathogen and highlights the bacterium’s virulence in humans. Awareness of individual anamnestic information and, in particular, possible contacts with domestic animals or recent consumption of unpasteurized dairy products was crucial for managing this outbreak. Equally important and of extreme value was the One Health interdisciplinary approach used to find solutions and solve community concerns. Further research is needed to gain insights into pathogenic characteristics of the strain responsible for the outbreak. Early diagnosis and identification of bacteria by using molecular methodologies will improve medical treatment outcomes, enable timely epidemiologic disease surveillance, and prevent spread of life-threatening infections.
